# 

**Published:** 1916-12-16

**Authors:** 


### CONTENTS.

| | PAGE |
|---|---|
| **Christmas in War-Time** ... ... ... ... ... | 211 |
| **Compulsory Latin for Medical Students** ... | 212 |
| **Hospital and Institutional News** ... ... ... | 213 |
| Memorial to Mr. Melhado—The Colour Bar at a London Hospital—Humanising Mechanical Treatments—The Power of the Specialist—The Germ of Scarlet Fever—General Medical Council Proceedings—An Exeter Small-pox Hospital Scheme—Nottingham Hospital and State Control—Municipal Gift to Coventry Hospital—More Hospital Troubles in New South Wales—Zeppelins and a Poor-Law Problem—An Extraordinary Source of Income—The "Future Decline" of Medical Practice—The David Lewis Colony Report—Boarding-out of Medical Staff—The Birth of Tuberculosis After-Care Associations—A Central Hostel for Army Nursing Probationers—Increased Salaries for Nurses—A Suggestion for State Pharmacies—This Week's Drug Market. | |
| **The New Hospital for Women** ... ... ... ... | 217 |
| Sir Alfred Keogh, K.C.B., on its Jubilee and Value. | |
| **Nature Studies in Macedonia** ... ... ... ... | 218 |
| **Hospital Music and Christmas Festivities** ... | 219 |
| **The Hospital Spirit in National Affairs** ... | 220 |
| **First Eastern General Hospital (T), Cambridge** ... ... ... ... ... ... ... | 221 |
| **Hospital Work in East Africa** ... ... ... ... | 222 |
| Kikuyu and Limoru. | |
| **The Matrons' and Sisters' Department** ... | 223 |
| Earning Power in Women Workers: A Momentous Opportunity. Round the Hospitals. The College of Nursing—Eight Reasons for Nurses—Matrons and Superintendents Co-operating—Australian and British Hospitals Contrasted—The Women of America. | |
| **Nurses' Pay** ... ... ... ... ... ... ... | 225 |
| St. Thomas's Hospital. | |
| **How the Russian Red Cross Gets its Wants Supplied** ... ... ... ... ... ... | 225 |
| **The Editor's Letter-Box** ... ... ... ... ... | 227 |
| British and Foreign Sailors' Society—The Eradication of Venereal Disease. | |
| **Humorous Medical Epitaphs** ... ... ... ... | 228 |
| **Hospital Needs and Hospital Appeals** ... ... | 229 |
| Hospitals with 150 Beds and Upwards—Hospitals under 150 Beds—Hospitals under 50 Beds—General Charities. | |
| **Christmas Number of the War Hospital Gazettes** ... ... ... ... ... ... | 233 |
| **Passing Events and Topics** ... ... ... ... | 233 |
| **Medical Appointments** ... ... ... ... | 233 |
| **The Trials of a Hospital Physician** ... ... | 234 |
| **Matrons' and Sisters' Appointments** ... ... | 234 |
| **Gifts and Bequests** ... ... ... ... ... | 234 |
| **The Institutional Worker** .. .. (Suppl.) | 1 |
| The Origin and Growth of a Workpeople's Fund. | |

